# Renal cell carcinoma of different pathological types in bilateral native kidneys of a kidney transplant recipient: A case report and literature review

**DOI:** 10.3389/fonc.2022.1112343

**Published:** 2023-01-16

**Authors:** Cheng Yi, Xiangyun You, Ang Sha, Zhen Zhang, Junfeng Yu, Xiaolin Guo, Henglong Hu

**Affiliations:** ^1^ Department of Urology, The First People’s Hospital of Yichang, China Three Gorges University, Yichang, Hubei, China; ^2^ Department of Urology, Tongji Hospital, Tongji Medical College, Huazhong University of Science and Technology, Wuhan, Hubei, China; ^3^ Department of General Surgery, Zhongxiang People’s Hospital, Zhongxiang, Hubei, China; ^4^ Department of Urology, Gushi People’s Hospital, Gushi, Henan, China

**Keywords:** renal cell carcinoma, kidney transplantation, bilateral renal tumors, native kidney, clear cell, papillary, nephrectomy

## Abstract

Patients after kidney transplantation have a much higher risk of developing malignant tumors than the general population. And the native kidney is an organ relatively susceptible to malignant tumors after renal transplantation. However, the simultaneous development of bilateral renal tumors is very rare; especially the bilateral native kidneys harbor different pathological types of renal cell carcinoma (RCC). We report a case of a patient who developed malignant tumors in both native kidneys nearly 19 years after renal transplantation. This patient underwent bilateral laparoscopic radical nephrectomy, and postoperative pathological examination showed clear cell RCC on the left native kidney and papillary RCC on the right one. And the early detection and surgical treatment resulted in a good prognosis. The literature related to the diagnosis and treatment of bilateral RCC after renal transplantation is also reviewed.

## Introduction

Renal transplantation is considered to be the best treatment for end-stage renal disease, which greatly improves the quality of life and prognosis of patients. However, due to the influence of the long-term use of immunosuppressive agents on the human immune system, renal transplantation recipients are more prone to malignant tumors (1). Renal cell carcinoma (RCC) is the third most common malignancy in renal transplant recipients after cutaneous tumors and hematopoietic cell diseases (2). However, the simultaneous presence of bilateral renal tumors is rare, especially in transplant recipients without autosomal dominant polycystic kidney disease. And the presence of bilateral primary renal tumors with different pathological components is more rare. In this paper, we described a renal transplant recipient who developed different pathological types of RCC in bilateral native kidneys.

## Case report

**Figure 1:**
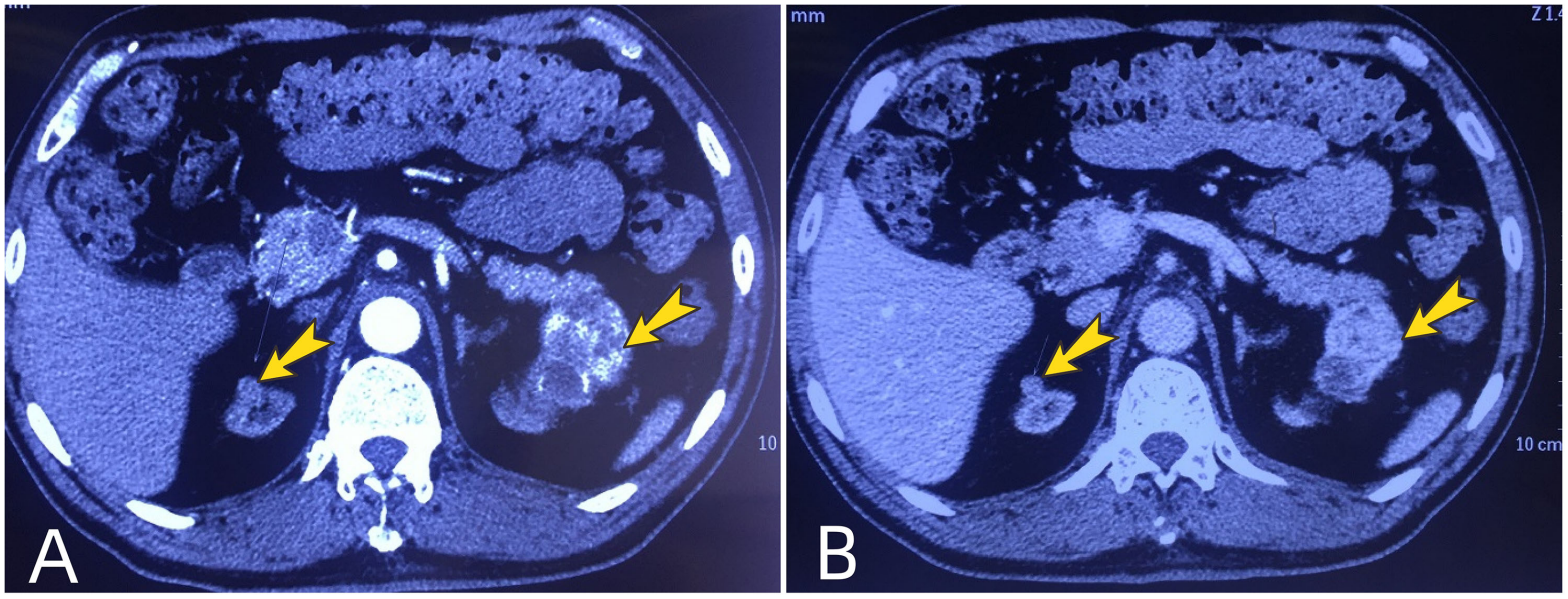
Preoperative renal enhanced computed tomography. Arrows show the tumors in the upper poles of bilateral native kidneys. **(A)** the tumors are enhanced in the cortical phase; **(B)** The enhancement of tumors is markedly reduced in the medullary phase, showing the performance of “fast in and fast out”.

The patient is a 61-year-old male. He underwent allogeneic kidney transplantation due to uremia in February 2000. The transplanted kidney was placed in the right iliac fossa. After the transplantation, he was normally on immunosuppressive therapy with cyclosporine (100 mg, 2 times/day) and mycophenolate mofetil (0.5 g, 2 times/day). And he maintained regular physical examinations and the function of his transplanted kidney was normal. A few years later, he developed symptoms of hypertension and started treatment with nifedipine controlled-release tablets (20 mg per night). He was also diagnosed with type 2 diabetes and was treated regularly with synthetic human insulin every day. In November 2018, he was admitted to a local hospital for poorly controlled diabetes. During this time, he underwent a Doppler ultrasound for both the native and transplanted kidneys. It showed that the left kidney had an extremely hypoechoic mass with a regular shape and clear boundary, and irregular anechoic areas could be seen in it. The size of the mass was 46 mm × 44 mm. Then an enhanced contrast CT examination of the abdomen and pelvis was performed. It showed that the mass was located in the upper pole of the left native kidney and it was significantly enhanced unevenly in the cortical phase, and the enhancement in the medullary phase was significantly reduced. Surprisingly, the CT revealed that the patient also had a nodule in the upper pole of the right native kidney and mild enhancement was seen in the nodule in the cortical phase (**Figure 1**).

**Figure 2:**
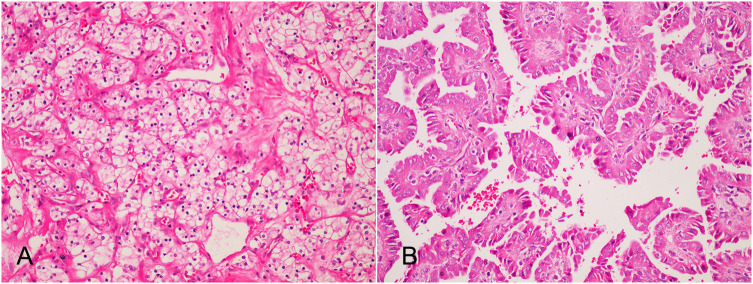
Postoperative pathology (HE×200): **(A)** The tumor in the left native kidney is clear cell renal cell carcinoma. The cancer cells are transparent and arranged in empty nests, and thin-walled sinusoidal vessels form a reticular septum; **(B)** The tumor in the left native kidney is type I papillary renal cell carcinoma of the right native kidney. Papillary structures formed by fibrovascular axis with foamy macrophages.

He was soon referred to our department with the diagnosis of bilateral native renal tumors. At the time of admission, he was in generally good condition. His height was 164 cm, and his body weight was 67 kg. His body temperature was 36.6°C and he has a heart rate of 80 beats per minute. His blood pressure was 189/104mmHg. A previous surgical scar was visible on the right lower abdomen. There was no percussion pain in the native kidney area, no tenderness in the ureteral area, and no abdominal mass was touched. No other significant positive signs were found. White blood cell count was 5.11×10^9/L, hemoglobin was 136.0 g/L, serum creatinine was 97 umol/L (reference values: 59 - 104 umol/L), eGFR was 72.3 ml/min/1.73m^2, fasting blood glucose was 6.55 mmol/L, urine protein was 2+, urine glucose was 1+. Chest X-ray and ECG showed no significant abnormalities. After proper preoperative preparation, the patient underwent bilateral laparoscopic retroperitoneal radical nephrectomy. The procedure was smooth and took 250 minutes. Postoperative gross specimens showed that both kidneys had marked atrophy of the renal cortex with a thickness of about 1 cm; a 43 mm × 40 mm gray-yellow and gray-red mass in the upper pole of the left kidney, with a cystic cavity in it; a gray-white nodule with a diameter of 12 mm in the upper pole of the right kidney, the cut surface was gray-white and showed a thin papillary shape. No signs of tumor invasion of the renal pelvis and hilar vessels were found in both kidneys. No lymph nodes were palpated in the hilar region. Pathological diagnosis (**Figure 2**): (left native kidney) clear cell RCC, WHO grade 1, T1bN0M0; (right native kidney) type 1 papillary RCC with papillary adenoma formation, Fuhrman grade 1, T1aN0M0. No tumor invasion of perirenal fat, renal sinus, or renal collecting system was found. No hilar lymph node metastasis was detected. The serum creatinine was 127 umol/L on the first postoperative day and it decreased to 112 umol/L on the fourth postoperative day. He recovered uneventfully and was discharged one week after surgery. During the one-year follow-up, he was alive without RCC recurrence and metastasis.

## Literature review and discussion

Kidney transplantation improves survival and quality of life for patients with end-stage renal disease and is less expensive than countless dialysis sessions, making it the preferred form of kidney replacement therapy (3, 4). Nevertheless, transplant recipients are at higher risk of developing malignancies and the renal cancer incidence is 6.8 times higher than that of the normal population due to long-term immunosuppression (5, 6). A recent meta-analysis showed that the overall estimated incidence of RCC was 0.7% in kidney transplant recipients (2). In addition, most renal cancer occurs in the native kidney, and only 9% of the tumors occur in the allograft itself (7). Clear cell carcinoma is the most common pathological type of RCC. However, the incidence of papillary RCC has increased among kidney transplant recipients. Some studies show that the incidence of papillary RCC after renal transplantation is up to 30% (8). The simultaneous occurrence of malignant tumors in bilateral native kidneys is rare, especially in patients without acquired cystic kidney disease. We identified only 13 cases in the published literature that have been shown in **Table 1** (9–18). The majority of these patients were male. The time interval between diagnosis and kidney transplantation ranged from 1 to 18.8 years, with a median time of 10.1 years. The case we report has the longest time interval. As can be seen from **Table 1**, unlike the conventional sporadic cases in which clear cell RCC is the predominant pathological type, the proportion of papillary RCC is markedly higher in native renal tumors after renal transplantation. As with patients with bilateral renal tumors in sporadic tumors, the pathological type of bilateral tumors were consistent in most patients. Interestingly, the case we present has clear cell RCC in the left native kidney and papillary RCC in the contralateral side, which is very rare.

**Table 1:** Published cases developing renal cell carcinoma in bilateral native kidneys after kidney transplantation.

First author year country	Sex	Age (y)	Years after transplantation	Immunosuppressive drug	Tumor size (cm) (left/right)	RCC types	Presenting signs	Stage (left/right)	Surgery	Follow-up time	Outcome
Carmellinil 1995 Italy (9)	male	45	4.8	cyclosporin + prednisone	5.0/5.0	granular cell carcinoma	intermittent fever episodes	T3	bilateral nephrectomy with lymphadenectomy	6 months	Died of RCC
Okumi 2001 Japan (10)	male	47	10.1	NA	1.6/3.3	alveolar type and granular cell subtype.	No	T1a	bilateral nephrectomy	30 months	AWRM
Ianhez 2007 Brazil (11)	NA	NA	NA	NA	3.0	papillary	NA	NA	bilateral nephrectomy	4 months	AWRM
Nobushita 2008 Japan (12)	female	26.5	8.5	methylprednislone + cyclosporin	4.0/4.5	clear cells	microscopic hematuria	NA	staged bilateral laparoscopic nephrectomy	18 months	AWRM
Klatte 2009 Italy (13)	male	58.7	3.3	NA	2.5/1.6	papillary	No	T1a	NA	5.1 years	Died of other cause
	male	38.6	15.3	NA	1.5/1.4	papillary	No	T1a	NA	4.6 years	AWRM
	male	46.7	11.6	NA	2.0/6.5	papillary	No	T1a/T1b	NA	6.7 years	AWRM
	male	45.9	14.1	NA	0.7/7.5	clear cells	No	T1a/T2a	NA	8.3 years	AWRM
Bing 2011 United States (14)	female	67	2	tacrolimus+mycophenolate+prednisone	1.1/2.2	clear cell papillary	NA	T1a	bilateral nephrectomy	21 months	AWRM
Cheung 2011 China (15)	male	48	12.3	cyclosporin+ prednisone	1.5/6.0	papillary	NA	T1a/T1b	bilateral nephrectomy	119 months	AWRM
Noce 2012 Italy (16)	male	61	4	mycophenolate mofetil+tacrolimus	10.0/3.7	NA	gross hemturia	T3	bilateral nephrectomy	NA	NA
Geramizadeh 2020 Iran (17)	male	16	6	methylprednisolone + cyclosporin	7.0/2.0	papillary	recurrent urinary tract infection	T1b/T1a	bilateral nephrectomy	3 years	AWRM
Hong 2021 China (18)	male	27	13	NA	0.3/2.5	papillary	No	T1a	staged bilateral retroperitoneal laparoscopic nephrectomy	3 months	AWRM
current case China	male	67	18.8	cyclosporin + prednisone	4.3/1.2	left: clear cell right: papillary	No	T1b/T1a	bilateral retroperitoneal laparoscopic nephrectomy	12 months	AWRM

AWRM, alive without recurrence and metastasis; NA, not available; RCC, renal cell carcinoma.

The exact reason for the increased risk of RCC after kidney transplantation is uncertain but is thought to be related to immunosuppression and loss of immunosurveillance (19). Risk factors for developing RCC after kidney transplantation include longer dialysis time before transplantation, history of acquired cystic kidney disease, smoking, male sex, older age, and hypertension (2, 20, 21). It is appropriate to perform screening of kidney tumors in post-transplant recipients, as they are prone to develop renal tumors, and early staged RCC often has no obvious symptoms. Ultrasound could play an important role in the detection of renal masses (22). European Association of Urology guidelines and some authors recommend an annual abdominal ultrasound screening to achieve an early diagnosis and a good prognosis (23, 24). In the case we present, the right kidney tumor was not detected at first by ultrasound. Therefore, computed tomography and/or magnetic resonance imaging are often needed as further confirmatory examinations, especially for patients with the renal cystic disease (25).

Localized bilateral native renal tumors can be treated with bilateral radical nephrectomy. Under appropriate circumstances, bilateral nephrectomy can be safely accomplished in a single procedure as in our case[13]. We performed a bilateral retroperitoneal laparoscopic nephrectomy for this patient and the operation is uneventful. Compare with the transperitoneal approach, the retroperitoneal approach is faster and allows extra-peritoneal dissection (26). During the operation, attention should be paid to the shape of the atrophic kidney and the anatomical changes of the renal pedicle blood vessels (27). The prognosis of localized native kidney RCC after nephrectomy is good (23). As shown in **Table 1**, most of the recipients with localized tumors were alive and without evidence of recurrence and metastasis during the follow-up period.

The treatment of advanced or metastatic RCC in renal transplant recipients can be challenging and the prognosis is relatively poor (28). Immunosuppression is a risk factor for tumorigenesis and progression, but interruptions and reductions in immunosuppressive treatment bring the risk of immunologic graft loss (29). There is currently no consensus on this dilemma. Reduction of immunosuppression drug dose and/or switch to mammalian target of rapamycin inhibitor, graftectomy, and complete withdrawal of immunosuppression, immune checkpoint inhibitors, or tyrosine kinase inhibitor are all possible options (30–32). Shared decision-making involving the urologist, oncologist, transplant specialist, and patient is essential to develop a rational treatment strategy.

In summary, transplant recipients are at higher risk of developing RCC, but simultaneous development of bilateral native kidney tumors is rare. Regular screening can be helpful in early diagnosis. Localized bilateral native renal tumors can be treated with bilateral radical nephrectomy and may have a good prognosis.

## Data availability statement

The original contributions presented in the study are included in the article/supplementary material, further inquiries can be directed to the corresponding author/s.

## Ethics statement

Written informed consent was obtained from the individual(s) for the publication of any potentially identifiable images or data included in this article.

## Author contributions

HH and CY conceived the project. XY and CY wrote the original draft.AS, ZZ and HH constructed the figure, revised the manuscript and performed the literature review. JY and XG assisted in the clinical case analysis and treatment, and literature review. All authors contributed to the article and approved the submitted version.
